# Advanced Collagen-Based Composites as Fertilizers Obtained by Recycling Lime Pelts Waste Resulted during Leather Manufacture

**DOI:** 10.3390/polym14153169

**Published:** 2022-08-03

**Authors:** Daniela Simina Stefan, Ana-Maria Manea-Saghin, Irene-Eva Triantaphyllidou, Ioanna Tzoumani, Irina Meghea

**Affiliations:** 1 Department of Analytical Chemistry and Environmental Engineering, Faculty of Applied Chemistry and Materials Science, University Politehnica of Bucharest, RO-011061 Bucharest, Romania; simina.stefan@upb.ro; 2 Research Center for Environmental Protection and Eco-Friendly Technologies, Faculty of Applied Chemistry and Materials Science, University Politehnica of Bucharest, RO-011061 Bucharest, Romania; 3 Department of Chemical Engineering, University of Patras, GR-26504 Patras, Greece; teva@upatras.gr; 4 Foundation for Research and Technology-Hellas (FORTH), Institute of Chemical Engineering Sciences (ICE-HT), GR-26504 Patras, Greece; tzoumani@upnet.gr; 5 Department of Chemistry, University of Patras, GR-26504 Patras, Greece; 6 Department of Mathematical Methods and Models, Faculty of Applied Sciences, University Politehnica of Bucharest, 313, RO-060042 Bucharest, Romania; irina.meghea@upb.ro

**Keywords:** collagen-based composites, leather waste recycling, advanced fertilizers, interaction mechanisms, agrochemical tests

## Abstract

Recent trends in ecological agriculture practices are focused on finding optimal solutions for reuse and recycling of pelt waste from tannery industry. In this context, new collagen-based hydrogels with NPK nutrients encapsulated have been functionalized with synthetic and natural additives, including starch and dolomite, to be used as composite fertilizers. Possible interaction mechanisms are presented in case of each synthetic or natural additive, ranging from strong linkages as a result of esterification reactions until hydrogen bonds and ionic valences. Such interactions are responsible for nutrient release towards soil and plants. These fertilizers have been adequately characterized for their physical chemical and biochemical properties, including nutrient content, and tested on three Greek poor soils and one Romanian normal soil samples. A series of agrochemical tests have been developed by evaluation of uptake and leaching of nutrients on mixtures of sand and soils. It was observed that the clay soil exhibits a higher adsorption capacity than the loam soil for most of nutrients leached from the composite fertilizers tested, with this being correlated with a slower control release towards cultivated plants, thus assuring efficiency of these collagen-based composite fertilizers. The most significant effect was obtained in the case of collagen-based fertilizer functionalized with starch.

## 1. Introduction

According to the principle of “circular economy”, most of the treatment techniques applied to waste mainly aim at their reuse and recycling while substantially reducing environmental pollution [1]. In this respect, recent strategies have been launched to treat organic waste in order to obtain valuable polymer composites to be recycled in industry and agriculture. Indeed, agriculture productivity can be much improved by using different organic and inorganic amendments, such as brown coals and natural zeolites [2], biochar and straw [3], bio-solids [4], organic compost produced from slaughterhouse waste [5], micro-algal biomass [6,7], farmyard manure and crops residues [8], etc.

These treatments are made in order to ameliorate the biological transformation of organic matter for obtaining a stabilized polymeric bio-composite avoiding any potential risks for plants and soils, while these products are applied as organic amendments. By adding such organic amendments as compost into the soil, major influences are exerted on the physical–chemical properties of the soil, able to modify the acids–bases relationships, with beneficial effects on increasing total cationic exchange and buffering capacities of the soils, as has been intensively studied during the last decades [9,10,11].

Our interest is focused on bovine leather recycling, which is generally directed towards obtaining protein composites by biochemical treatments using microorganism enzymes and obtaining protein hydrolysates and protein binders with different uses. Such organic biopolymers are an important source of raw materials for agriculture, as the protein waste matrix provides sufficient elements to improve the composition of poor and degraded soils, and many plants can benefit from elements such as nitrogen, calcium, magnesium, sodium, and potassium [12,13].

A way to valorize the untreated fleshing and trimming, bovine hide waste is the product of a three-dimensional molecular network named hydrogels by cross-linking the proteins hydrolyzed with polymers based on polyacrylamide, polyvinyl alcohol, oligo oxyethylene methacrylate, acrylic acid, maleic acid, cellulose, starch, and gum, which form three-dimensional molecular networks. The hydrogels enriched with nutrients C, N, P, and K can be used as efficient amendments in agriculture for degraded soils [14,15].

Such an approach meets the stringent demand to counteract the decline of soil fertility and productivity, which is in line with growing interest in the general improvement of the quality of soils by adding organic amendments from different sources [16,17].

Indeed, for ecological reasons, other solutions for reuse and recycling of pelt waste from the tannery industry have been proposed, one of them being for collagen hydrolysate with micronutrients incorporated to be used as fertilizers for poor soil’s rehabilitation [18,19,20].

Our previous works [21,22,23] reported on the synthesis and physical–chemical characterization of novel collagen-based hydrogels by recycling the protein hide waste from the leather industry in ecological conditions and obtaining advanced collagen-based composites with N, P, and K nutrients encapsulated and functionalized with two synthetic polymers, namely poly-acrylamide and poly(sodium 4-styrenesulfonate-co-glycidyl methacrylate) (P(SSNa-co-GMAx), and two natural compounds, namely starch and dolomite, in order to be used as fertilizers for poor soil amendment. The reason why the last two natural compounds were selected for collagen functionalization was to test their efficacy on improving fertilizer quality by using starch as an organic and dolomite as a mineral amendment, as they are both available in high amounts and as waste to be recycled.

The current work aims at testing these new biocomposite fertilizers for their nutritive characteristics and nutrient-leaching properties. In this respect, detailed study of the physical–chemical and biochemical characterization of both fertilizers and soils was conducted in order to establish their biocompatibility and possible interactions. For each additive encapsulated, the intercalation mechanisms were analyzed and correlated with the uptake and leaching of nutrients on sand and soils. The beneficial effects in improving soil quality were proven by testing the biological amelioration of soil fertility.

## 2. Materials and Methods

### 2.1. Materials

Multipolymeric collagen-based agro-hydrogels were prepared, using as raw material the limed hide waste (no haired) from fleshing and trimming bovine hides (lime fleshing), which was provided by SC PIELOREX tannery, Jilava, Ilfov county, Romania. Collagen hydrolysate was obtained according to our previous studies [24,25]. Briefly, gelatin hide was subjected to acid hydrolysis in presence of potassium phosphate, and the protein hydrolysate was functionalized with 5% synthetic and natural polymers or mineral waste, such as poly-acrylamide, poly(sodium 4-styrenesulfonate-co-glycidyl methacrylate) (P(SSNa-co-GMAx), starch, or dolomite, in order to obtain efficient composite fertilizers.

It is obvious that during acid hydrolysis for obtaining collagen hydrogel, the calcium content provided by lime is almost completely removed, and its remnant contribution will have not a significant effect on the composite quality, as the fertilizers will be applied for poor soils amendment, and these usually have carbonates (circa 30–50 mg/kg) as calcite and dolomite in their mineralogical composition.

For the studied samples, the following codes were allocated:

CH—collagen hydrolysate,

Ref-CH—collagen hydrolysate with phosphorus and potassium nutrients encapsulated as reference sample,

PSSG—Ref-CH functionalized with 5% P(SSNa-co-GMAx) copolymer,

POLY—Ref-CH functionalized with 5% poly-acrylamide,

AMI—Ref-CH functionalized with 5% starch,

DO—Ref-CH functionalized with 5% dolomite.

### 2.2. Physical–Chemical Analyses of Soils, Leachate, and Harvested Crops

Physical and chemical analyses of soils were performed as follows:

pH was determined by potentiometric method in aqueous suspension of soil:water ratio 1:2.5, with glass-calomel combined electrode; the potentiometer was calibrated with buffer solutions of known pH prior to the analysis of samples.

Organic matter (humus), OC%, was determined according to STAS 7184/21-82, volumetric method, and by wet oxidation modified Walkley–Black method.

Total nitrogen (TN%) was determined by Kjeldahl method: digestion with H_2_SO_4_ at 350 °C and potassium sulphate and copper sulphate catalyst—SR ISO 11261:2000.

Total content of soluble salts (extraction ratio 1:10)—STAS 7184/7–87; SR ISO 11265 + A1:1998.

Exchangeable (accessible, mobile) phosphorus (EP%) followed these steps: extraction according to Ègner–Riehm–Domingo procedure and was then determined by spectrophotometry (CINTRA 404 UV–VIS spectrometer) with molybdenum blue after reduction with ascorbic acid [26].

Exchangeable (accessible, mobile) potassium (EK%) was obtained by extraction after Ègner–Riehm–Domingo procedure and then determination by flame photometry (Flame Photometers S-935).

Alkaline soil carbonates of the air-dried soil fraction (particle size < 2 mm) were estimated by calculation of measured carbon dioxide from carbonates decomposed with hydrochloric acid (1:3)-STAS 7184/16–80, total phosphorus by spectrophotometry (CINTRA 404 UV–VIS spectrometer), and total potassium by flame photometry (Flame Photometers S-935) [27].

### 2.3. Biochemical Analysis of Soils

Soil samples considered as poor soils were collected from three different regions of western Greece: Kernitsa Achaias (S1), Neochori Messolonghiou (S2), and University Patras (S3). For comparison purposes, a fourth sample of normal soil was collected from Aldeni, Buzau, Romania (S4). They belong to the following textural classes: S1—loam soil (S1-L), S2—clay loam soil (S2-CL), S3—silty clay loam (S3-SiCL), and S4—sandy clay loam (S4-SCL). The fertility state of poor and normal soils was assessed by the function of their nutrient content; indeed, for the samples S1–S3, extractible phosphorous was below detection limit, while extractible potassium was below 50 mg/kg K_2_O; this means a low-to-reduced fertility state. In contrast, for soil S4, phosphorous was present in the amount of 21 mg/kg P_2_O_5_ and potassium 144 mg/kg K_2_O, with such values corresponding to a high fertility state (>20 mg/kg P_2_O_5_ and >80 mg/kg K_2_O).

All samples were collected in sterile containers at a depth of 10 to 30 cm and stored at 4 °C in the dark for no more than 48 h prior to microbial examination. Serial dilutions of soil samples were made, followed by sterile deionized water addition in order to achieve 10^−1^ to 10^−9^ g of soil/mL suspensions. In every following technique described, the proper dilutions within this interval were chosen in order to be applied on the selective media, and the developed microorganisms are expressed as colony-forming units per gram (CFU/g). Each experiment was conducted in triplicate.

The number of CFU of cultivable aerobic mesophilic bacteria was performed by standard plate counting method: 1 mL of the chosen diluted samples, prepared as described above, were added to a Petri dish plate (9 cm) and followed by the addition of 15 mL of Plate Count Agar (PCA) (Condalab, Madrid, Spain), which was prepared according to manufactures instructions. Plates were then incubated aerobically for 24 to 72 h at 30 ± 1 °C. In order to determine the number of CFU of cultivable proteolytic bacteria present in the soil samples, the proper dilutions were counted onto Plate Count Agar supplemented with 10 g/L of skimmed milk (PCA-SM) (Condalab, Madrid, Spain) in Petri dishes (9 cm). Plates were incubated aerobically for 24 to 72 h at 30 ± 1 °C. The number of CFU of cultivable soil yeasts and molds (fungi) was determined using the proper dilutions of the soil and spread on Petri dishes (9 cm) containing 20 mL of Rose Bengal Chloramphenicol Agar (PBCA) (Condalab, Madrid, Spain), which was prepared according to the manufacturer instructions. All plates were incubated aerobically for 3 to 5 days at 20.5 ± 1 °C. To determine the number of CFU of cultivable actinomyces in the soil samples, the proper dilutions of soil were used in order to inoculate Petri dishes (9 cm) containing 20 mL of Sheep Blood Agar (Condalab, Madrid, Spain), which was prepared according to the manufacturer instructions. All plates were incubated in anaerobic conditions for 3 to 7 days at 35 ± 1 °C.

Microbial biomass of Romanian soil S4-SCL amended with fertilizer was determined by indirect methods, such as substrate-induced respiration and fumigation extraction methods [28], according to SR ISO 14240–2001, Part 1 and Part 2.

Evaluation of functional bacteria of soil microorganisms was made by CLPP method: Community Level Physiological Profiling [29].

### 2.4. Evaluation of Exchangeable NPK Nutrients of Agro-Hydrogels in Soils

**Method.** Soil poor in nutritive elements sampled from the western Greece region was placed in an aluminum vessel and mixed with the agro-hydrogel as liquid, dried, or gel in various final proportions: 1%, 5%, 10%, 30%, and 50%. Portions of these mixtures were sampled at different time intervals (e.g., 1, 3, 7, 14, 21, and 30 days) and analyzed for ammonium leaching. Changeable ammonium could be evaluated by elution of the soil mixture with a solution 2.0 M KCl, and ammonium nitrogen was determined by a modified Kjeldahl method. The experiments were performed in triplicate. On the graphs are shown the average values. The standard deviation was between ±5%.

### 2.5. Determination of Leached Ammonium and Phosphate Ions

The agrochemical tests for characterization of fertilizers with slow release and determination of their percolation degree in soils are mainly defined by SR EN 13266/2002—Fertilizers with slow dissolution rate—Determination of nutrients leaching and SR CEN/TR 14405/2009—Waste characterization. Behavioral tests on leaching. Percolation test in ascendant counter-flux.

**Method.** An amount of 5 g of each fertilizer tested was completely dissolved into 5 L distilled water and transferred through a column. A constant elution flux of 225 mL/h was assured for each fertilizer. Volumes of 200 mL of leaching were collected and analyzed for their ammonium, nitrate, and phosphate ions contents. Experiments were performed at 25 °C in triplicate.

## 3. Results

### 3.1. Characterization of Compounded Hydrogels with Nutrients Encapsulated

Physical–chemical characterization of composite agro-hydrogels is given in Table 1 and Table 2.

The fertilizer quality is mainly conferred by the content of NPK nutrients available for plant growth and their leaching in soil solutions. The absolute values in nutrient content are reflected by the elemental analysis data given in Table 1.

According to these data, one may observe that all the fertilizers exhibits quite similar chemical composition, as functionalization with 5% synthetic or natural additive does not result in a major change in proportion of NPK content, with this corresponding to the general chemical formula N_10_P_6_K_10_, which is significant for agriculture application.

After the preliminary agrochemical tests, a special attention was directed to the collagen hydrogels functionalized with natural compounds, such as starch (AMI) and dolomite (DO), which fulfilled both requirements referring to fertilization efficiency and those related to economic aspects, as these are available as waste and are therefore cost-effective. In this respect, the agrochemical characteristics of AMI and DO fertilizers were determined by values of nutrient content soluble in water, expressed by total nitrogen, phosphorus, potassium, and total content of soluble salts. The values of pH and electrical conductivity were determined in aqueous suspensions of various concentrations ranging between 0.5% and 10%. The results are collected in Table 2, and the methods used are in accordance with European Commission Regulation 2003/2003, adjusted due to the organic matrix of these fertilizers, and also in accordance with procedure described in [22].

### 3.2. Biochemical Characterization of Soils

The data on the microbial analysis of Greek soils by standard plate counting method are available in the Table 3.

As can be seen from Table 3, the number of microorganisms in samples S1-L-S3-SiCL is at the level of magnitude order of 10^6^, which proves a low microbial activity, with these soils being poor in organic compounds.

For a normal soil sample, special microbiological tests were conducted in order to prove the biological amelioration of soil fertility while applying the best-performant collagen hydrogel functionalized with starch. These experiments were performed on a sandy clay loam soil (S4-SCL-Aldeni-Buzau, Romania), having as reference sample the moist soil as compared with two samples of soil humectated with aqueous suspensions 0.1% and 0.2% of AMI hydrogels. The main biologic indicators are collected in Table 4.

This normal soil sample (S4-SCL) exhibits an initial number of microorganisms at the magnitude order of 10^9^, being a fertile soil rich in biodegradable organic compounds when compared with data from Table 3 for the three poor soils, which are characterized by a number of microorganisms of three magnitude orders lower.

### 3.3. Agrochemical Tests of New Fertilizers on Soils

Tests on leaching ammonium and phosphate ions from composite fertilizers were performed on columns filled with gravel and sand in one series of experiments and in another series on columns filled with gravel and a mixture of sand with each of two Greek soils (80 g sand and 20 g soil from samples S1-L and S2-CL). The initial content in nitrogen and phosphorus as water-soluble ions in the fertilizers tested, Ref-CH, AMI, and PSSG, are as follows: 426.5, 363.75, and 588.5 mg N/g and 75, 85, and 83 mg P/g, respectively.

**Figure 1:**
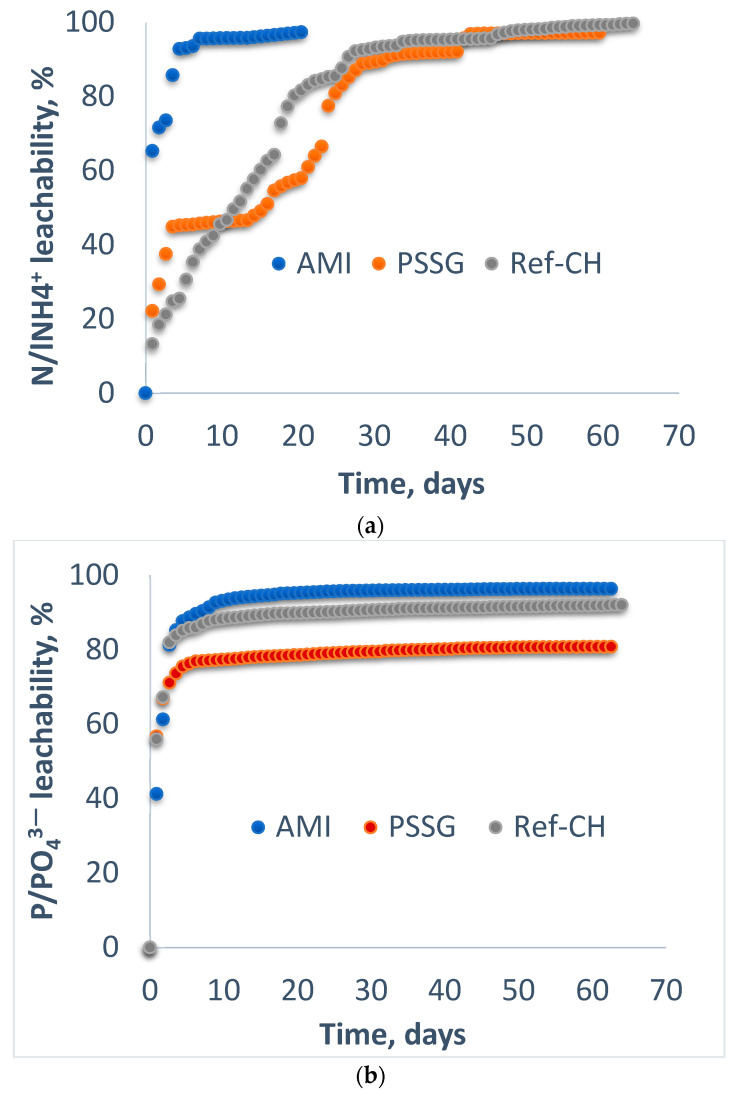
Leachability of ammonium nitrogen (**a**) and phosphorus (**b**) from fertilizers on sand column.

The evolution in the time of ions leaching as ammonium and phosphate on columns with gravel and sand is illustrated in Figure 1a,b.

From the analysis of Figure 1a, one may observe that in the case of the AMI fertilizer based on starch, an amount of 95% of nitrogen as ammonium leached during the first 7 days, while the remainder of 3% as released during next 13-day period.

In case of the PSSG fertilizer, four zones can be distinguished. One zone of rapid release was in the first 2 days when circa 45% of ammonium nitrogen leached, followed by a slow-release zone of 2% during the next 10 days, with the third zone being once again a rapid release of circa 43% during 20 days, and the remaining nitrogen was slowly released with a rate of 3% per day. The difference between the ammonium-releasing rate of these two fertilizers is in accordance with stronger covalent bonds established in case of synthetic polymer PSSG, when there are some esterification reactions involving various functional groups of the collagen chains, while in the case of starch, the hydrogen bonds are mainly responsible for the affinity of the bio-fertilizer components, as will be discussed in the next section.

Reference fertilizer Ref-CH not functionalized has a relative constant rate of ammonium nitrogen leaching after 2–3 days, when 20% of nitrogen was rapidly released; during the next 28 days, the rate of release was 2%/day, while in the last 40 days, the release slowed down to 2%/day.

In contrast to ammonium nitrogen release, in the case of phosphorus, the leaching behavior is quite similar for the three fertilizers tested on sand: with very rapid release during the first five days, when over 90% of phosphorus was released by Ref-CH and ***PSSG*** fertilizers and over 95% in case of AMI. In the next 60 days, the rest of the phosphorus was released, until 98% in case of AMI, 92% for Ref-CH, and 80% for *PSSG*. This similar behavior of the three fertilizers can be explained by similar structures of these composites, of which the polymers are encapsulated inside the hydrogel matrix between two collagen chains, while the potassium phosphate groups remain outside the capsule, facilitating their easier release towards soil and plants.

The above experiments can be relevant for the sandy soil sampled from Romania (S4-SCL).

Similar experiments were carried out for leaching ammonium and phosphate ions of these fertilizers on columns filled with a mixture of sand and two of the tested poor soils (S1-L and S2-CL) in a proportion 4:1 of sand to soil.

**Figure 2:**
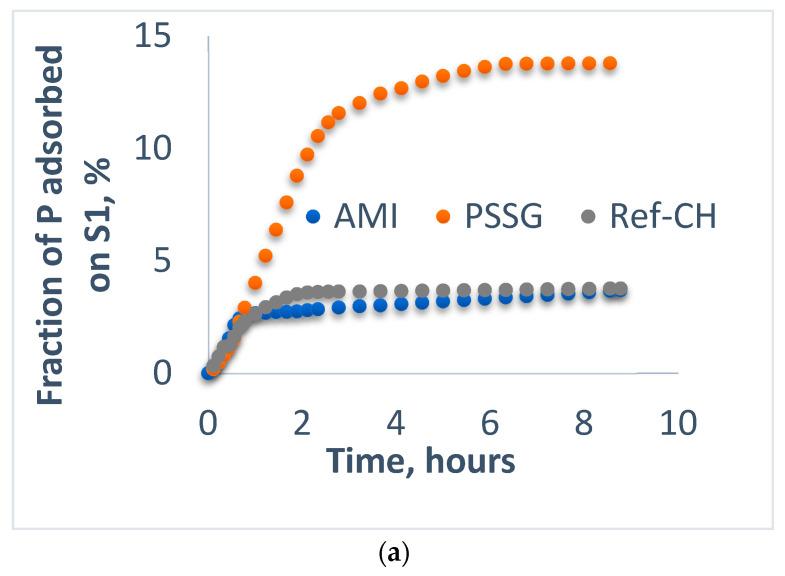

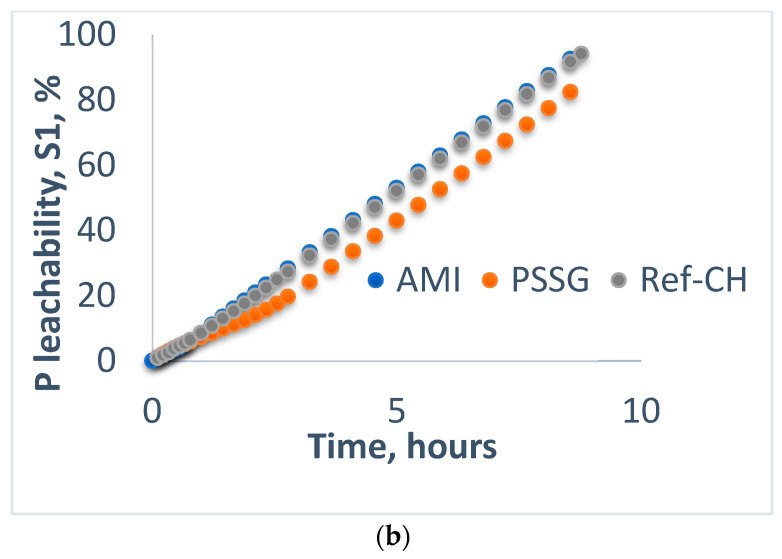
Fractions of phosphorus adsorbed (**a**) and leached (**b**) for the fertilizers on sand and soil S1-L.

**Figure 3:**
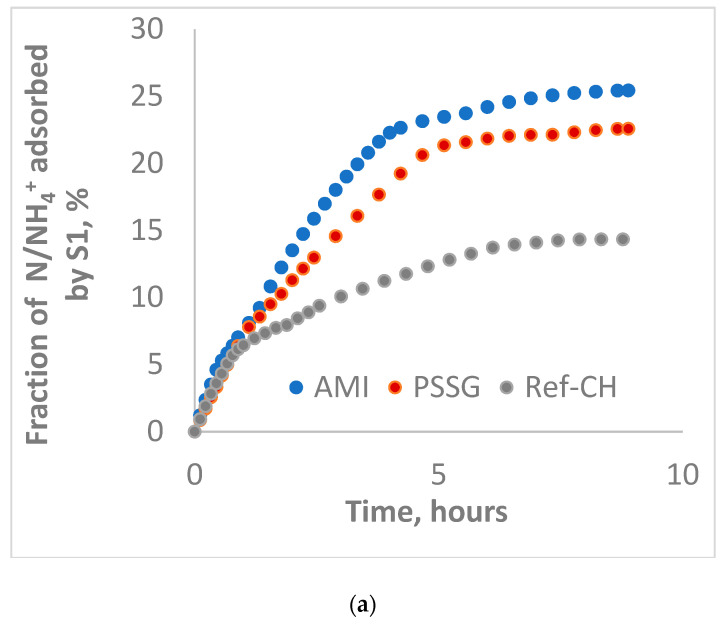

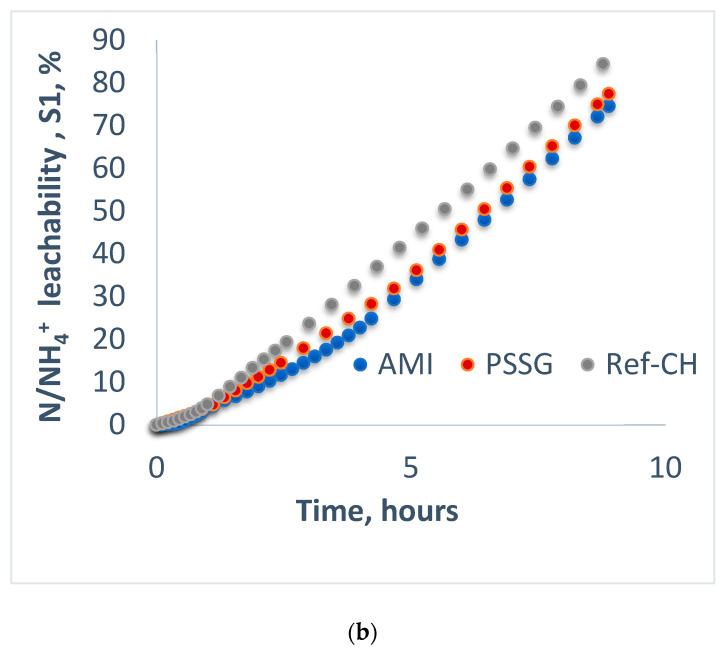
Fractions of ammonium adsorbed (**a**) and leached (**b**) for the fertilizers on the mixture of sand and soil S1-L.

**Figure 4:**
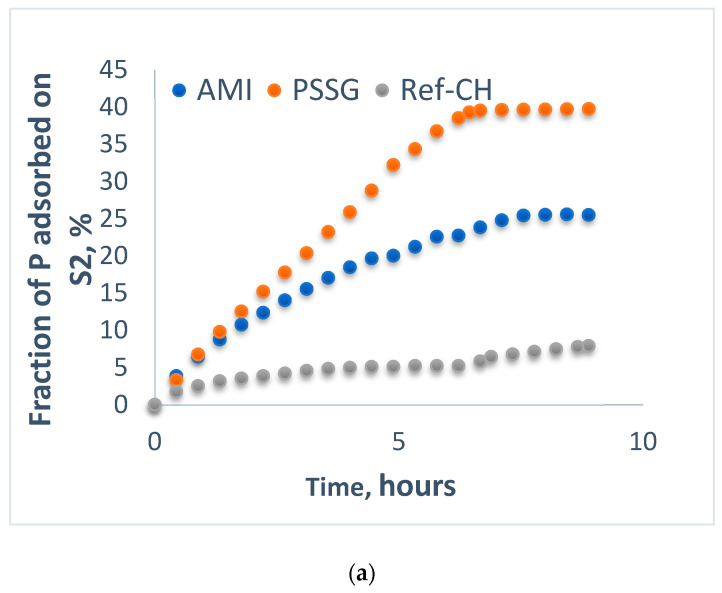

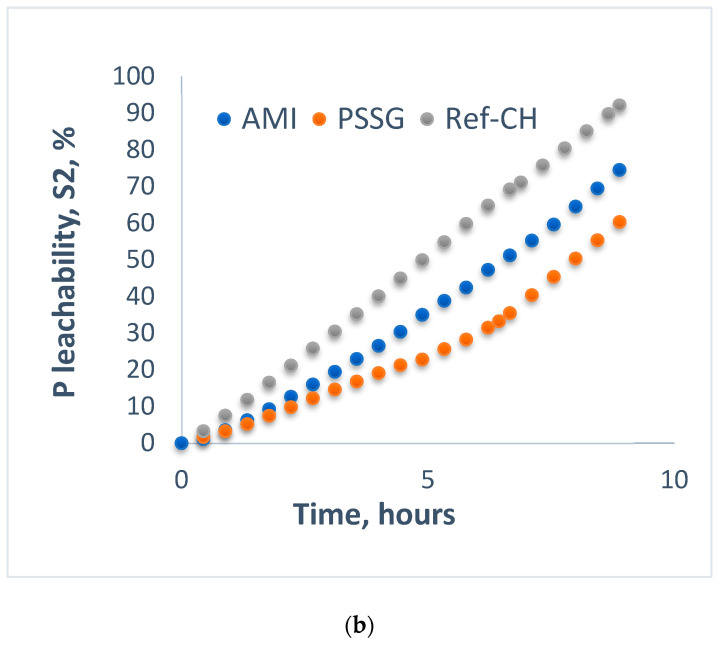
Fractions of phosphorus adsorbed (**a**) and leached (**b**) for the fertilizers on the mixture of sand and soil S2-CL.

**Figure 5:**
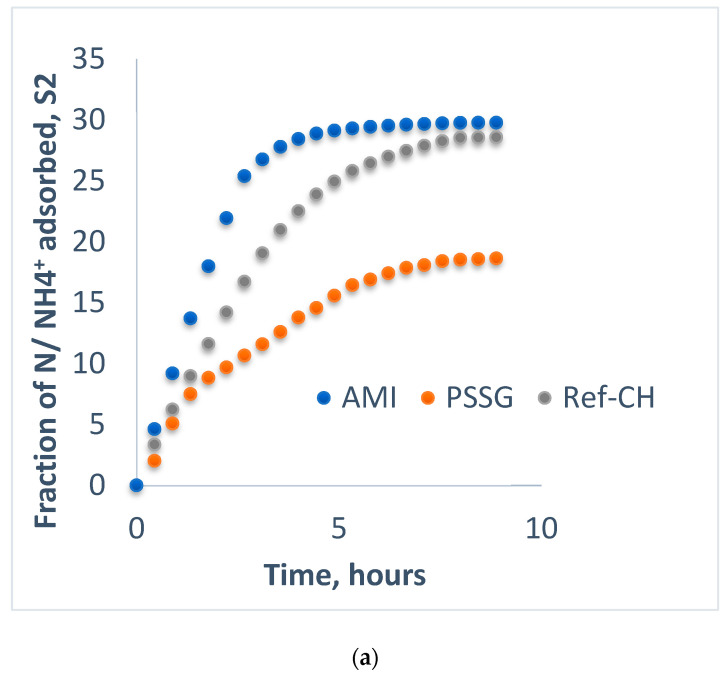

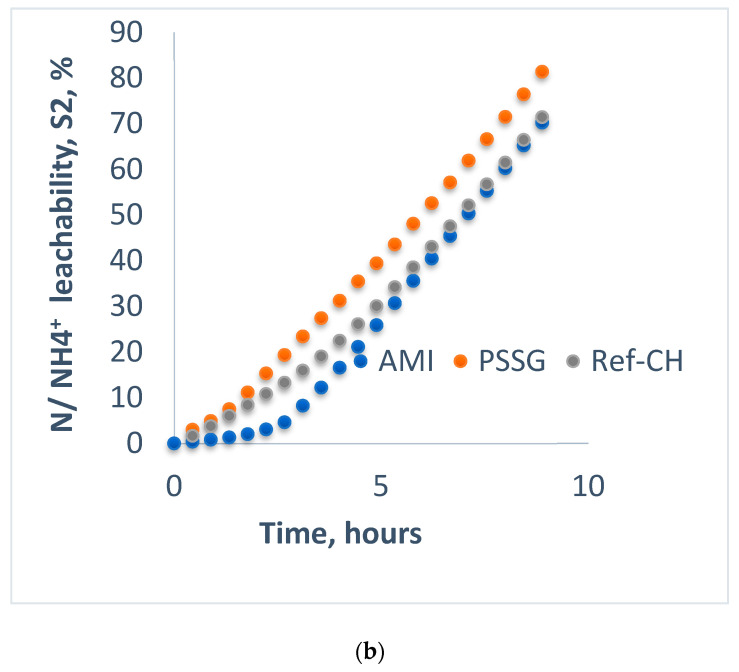
Fractions of ammonium nitrogen adsorbed (**a**) and leached (**b**) of the fertilizers on the mixture of sand and soil S2-CL.

Aliquots sampled from solutions of each fertilizer were analyzed before (fraction adsorbed) and after passing (fraction leached) through the columns filled with mixtures of sand and soils S1-L and S2-CL, respectively, and the data were graphically represented in Figure 2a, Figure 3a, Figure 4a, Figure 5a and Figure 2b, Figure 3b, Figure 4b, Figure 5b for the fractions adsorbed and leached, respectively, while the most representative events during their evolution in time are collected in Table 5.

From Figure 2a, one may observe that the soil S1-L reached the maximum adsorption capacity of phosphorus after one hour in the case of the AMI fertilizer, after 2 h for Ref-CH, and after 6 h for *PSSG*, while for ammonium nitrogen, the adsorption capacity reached its maximum values after 6 h for all three fertilizers tested (see Table 5).

Concomitantly, the leachability of both phosphorus and ammonium continuously increased until their exhaustion for all fertilizers on both soils (Figure 2b, Figure 3b, Figure 4b and Figure 5b).

## 4. Discussion

### 4.1. Physical–Chemical Interactions Inside Polymeric Composites

The main structural transformations produced during the process of extraction of collagen by acid hydrolysis from leather waste are discussed in the reference [30]. The triple helix of collagen is further unfolded in the steps of obtaining of hydrogel, when numerous functional groups are activated to assure high affinity of the new polymeric composites. The main step in this hydrolysis process is functionalization with adequate nutrients to confer soil fertility. While the nitrogen content is assured by the amine and amide groups of collagen hydrolysate, potassium and phosphorous are acquired by final hydrolysis with K_2_HPO_4_, when potassium phosphate anions are attached at polymeric chain by means of carbonyl groups.

One may expect that the new polymer composites may have proper fertilization qualities, as phosphorus and potassium nutrients will be easily available to be transferred into the soil and then towards plants since they are ionic species obtained by encapsulating dipotassium phosphate salt into the collagen matrix. However, the nitrogen is available as covalently linked into protein compounds and should be more difficult to biodegrade into exchangeable ammonium species, the best source of nitrogen for plants. For this reason, an indirect method was proposed for testing the availability of ammonium nitrogen for plants.

The structure of composite fertilizers as determined by the active groups on gelatin hydrolysate, such as amine, carbonyl, carboxyl, hydroxyl groups, etc. The possible reactions and structures of new composite fertilizers functionalized with poly-acrylamide and poly(sodium 4-styrenesulfonate-co-glycidyl methacrylate) (P(SSNa-co-GMAx-PSSG) and two natural compounds, starch and dolomite, are presented in Table 6. One may observe that in all the cases, the functionalized agent is interspersed between collagen hydrolysate chains, but the interaction mechanisms are different, resulting in linkages of various strengths. Thus, in case of synthetic polymers poly-acrylamide and PSSG, dominant interactions can be assessed as esterification and amidation reactions, leading to rather strong covalent bonds between the additive and collagenous matrix. While polyacrylamide has many reactive groups able to link the collagenous chains in a double-stranded coil manner by means of carboxyl, hydroxyl, carboxyl, and di-imine groups, in the case of PSSG, only marginal carboxylic groups are available for esterification, thus keeping the collagen chains at a greater distance. However, in both cases, the polymeric additive seems to be encapsulated inside the collagen matrix, while potassium phosphate groups remain outside the capsules, thus being available for ionic exchange during the fertilization process.

Completely different types of interactions are established during encapsulation of starch and dolomite into hydrogel matrix. The highly saturated structure of natural polymer starch, dominated by α1–4 glycoside linkages along linear chains of amylose and α1–6 glycosidic linkages at branch points in amylopectin, confers a lower reactivity with collagen groups. In this way, higher compatibility between the two biopolymers is assured by means of numerous hydrogen bonds established by glycoside groups of starch with hydroxyl and amino groups of collagen, while only a few carboxyl groups can be involved in esterification reactions. Such rather low- or medium-strong bonds add to this biopolymer composite enough stability when is applied on the soil and at the same time assure an easy release of NPK nutrients together with additional biodegradable organic carbon content.

When dolomite is encapsulated within NPK collagen hydrogel, the composite consistency is mainly assured by means of hydrogen bonds between carbonate oxygen and hydrogenated groups of hydrogel, such as carboxyl and hydroxyl, but some ionic interactions caused by calcium and magnesium cations could not be neglected, which could facilitate the ionic exchange toward soil and plants.

These interaction mechanisms will provide the theoretical support for further interpretations of the specific behavior of these new composite fertilizers on uptake and leaching toward various types of soils.

### 4.2. Behavior of Fertilizer Nutrients during Uptake and Leaching on Sand and Soils

Improving soils’ fertility by adding various amendments containing organic components is mainly analyzed by means of mineralization of carbon and nitrogen [34] and their controlled release [35].

In this context, a comprehensive study was performed on these new agro-hydrogels referring to their biodegradability in aerobic conditions in water and in composting environment, completed by the leaching of the ammonium, nitrate, and phosphate ions on columns filled with gravel and sand mixed with each of poor soils tested.

The big difference noticed between the leaching rates of the ammonium and phosphorous nutrients can be explained by the special structure of these composite fertilizers, which contain phosphorus and potassium as ionic species incorporated into collagen hydrolysate matrix as a result of encapsulation with dipotassium phosphate, while the nitrogen is available in the covalent form of protein compounds and needs several degradation steps to be transformed from organic into inorganic species as nitrate and ammonium.

A comparative analysis of adsorption isotherms from Figure 2a, Figure 3a, Figure 4a and Figure 5a provides the values for maximum adsorption capacity (A_max_) and time to reach the equilibrium on the two tested soils for phosphorus and nitrogen nutrients (Table 5).

In most of cases, the average time for reaching adsorption equilibrium was 6–7 h except for reference fertilizer (Ref-CH) and that functionalized with starch (AMI) for phosphorus as a result of its encapsulation in ionic form, as it is a more easily exchangeable species than nitrogen, as explained before. At the same time, the leaching period was quite similar in all the cases, around 8–9 h, which is correlated with nutrient control leaching from soil to the plants. These time intervals in hours seem to be rather short, but we should take into account that these laboratory experiments on columns were rather accelerated, as only a proportion of 4:1 sand to soil was used. It is obvious that in real agriculture conditions in the soils, these time intervals will be much longer, in accordance with vegetation period of plants, as it was also established by Anghel et al. [35].

Another useful remark after analysis of adsorption capacities values from Table 5 is related to the dominant concentrations of ammonium nitrogen in all the cases studied, as this is in accordance with initial nutrient content of the fertilizers previously presented in Section 3.1, and their atomic ratio of N_10_P_6_K_10_ was calculated using elemental analysis data from Table 1; these are values that recommend these collagen-based composites as important source of protein origin nitrogen for amendment of poor soils. These data are in agreement with the results obtained by Lima et al. [36] for the use of leather waste as a nitrogen source for plant growth.

When comparing the two poor soils analyzed here, one may notice that the clay soil (S2-CL) exhibits a higher adsorption capacity than loam soil (S1-L) for most of the nutrients released from the fertilizers tested, and this higher adsorption capacity on soil could be correlated with a slower control release towards cultivated plants, thus assuring the efficiency of these composite fertilizers.

### 4.3. Biological Amelioration of Soils’ Fertility

Laboratory tests emphasized the beneficial effects on soil amelioration provided by protein structure of the collagen hydrogel functionalized with starch (AMI) on some biologic indicators, as shown in Table 4.

For the soil samples treated with AMI fertilizer suspensions, the number of CFU of cultivable mesophilic organotrope bacteria was significantly increased in both concentration variants by 61–81%, indicating the stimulation of development of this taxonomic group. Simultaneously, there was an increase of species diversity belonging to *Bacillus* strain by developing some species attesting to the improvement in soil humidity and aeration, such as *B. subtilis* and *B. egaterium*. Frequently, some species were isolated from the zymophenic flora belonging to the *Pseudomonas* strain.

The number of CFU cultivable mesophilic fungi, with a decreasing tendency, does not significantly differ as a function of treatment. In both variants, there is a number of 7–9 similar species, the most dominant being the *Aspergillus* strain, accompanied by *Cephalosporium* and *Penicillium*. The *Cladosporium herbarum* strain, with a role in aggregating soil particles, was isolated in both fertilization variants with protein biopolymers based on collagen hydrolysate and functionalized with starch and dolomite.

Physiological activities of microflora, expressed by the amount of CO_2_ released during respiratory processes of soil, were also influenced for a 5% probability by the treatments applied, which showed an increased evolution.

The microbial biomass significantly increased by 19% for the variant with 0.1% hydrogel AMI against reference soil, with this value being mainly supported by the bacterial component of soil microflora, which assures intensification of metabolic activities by means of CO_2_ release. This amendment variant applied for a sugar beet subculture on this sandy clay soil assures an important multiplying effect of microbial mass and soil respiration together with an equilibrated microscopic bacteria/fungi balance.

One may conclude that the use of AMI collagen hydrogel with encapsulated starch for poor soil structure amelioration positively influenced the micro-colonies in soil, creating adequate conditions for maintaining strain diversity and their equilibrium balance and thus enhancing their physiologic activities as a result of improving the biophysical properties of soil.

## 5. Conclusions

A systematic study is presented on reusing hide waste resulting from the leather industry by capitalizing on their valuable protein components to obtain efficient collagen-based hydrolysates as composite fertilizers for poor soils’ amelioration. A series of polymer composite fertilizers was obtained by the functionalization of collagen hydrolysates with some synthetic and natural compounds after encapsulation with P and K nutrients. Two representative organic and mineral amendments, starch and dolomite, were selected, as both of them are available in high amounts as waste to be recycled.

Possible interaction mechanisms during encapsulation of nutrients and synthetic or natural polymers/ore are discussed in tight connection with their uptake and leaching on studied soils and the effects on improving soil fertility.

All the composite fertilizers tested exhibited a similar composition in atomic ratio of N_10_P_6_K_10_, which is significant for agriculture applications.

The study of nutrient leaching on soils S1-L and S2-CL revealed that the soil adsorbs nitrogen and phosphorus as ammonium and phosphate ions, respectively.

The initial number of microorganisms in three poor soils was at the level of magnitude order of 10^6^, which proves a low microbial activity, with these soils having a low organic content, while for a normal soil, the level of magnitude order of 10^10^ proves a high microbial activity in accordance with its high fertility.

Finally, it was demonstrated that the use of collagen hydrogel with encapsulated starch for poor soil structure amelioration positively influenced the micro-colonies in soil, creating adequate conditions for maintaining strain diversity and their equilibrium balance and thus enhancing their physiologic activities as a result of improving the biophysical properties of soil.

## Figures and Tables

**Table 1:** Nutrient content in tested composite fertilizers.

Chemical Analysis, %	Ref-CH	PSSG	POLY	AMI	DO
**Nitrogen (N)**	9.91	9.86	10.70	9.90	9.89
**Phosphorus (P)**	5.50	5.20	5.25	5.30	5.22
**Potassium (K)**	10.07	9.55	9.50	9.28	9.95

**Table 2:** Nutritive characteristics of hydrogels functionalized with starch (AMI) and dolomite (DO).

			Initial Fertilizers		Concentration in Suspension							
Nr. Crt.	Parameter	Units	AMI	DO	0.5%		1%		5%		10%	
					AMI	DO	AMI	DO	AMI	DO	AMI	DO
**1**	**Total nitrogen (Nt)**	%	6.97	10.13								
**2**	**Total phosphorus (P_2_O_5_)**	%	1.50	3.43								
**3**	**Potassium, water-soluble (K_2_O)**	%	21.97	2.35								
**4**	**Total content of soluble salts, extraction ratio 1:10**	g/100 g	32.80	21.91								
**5**	**pH solution conc.**	pH units			7.60	3.70	7.55	3.63	7.43	3.50	7.37	3.43
**6**	**Conductivity solution conc.**	mS/cm			3.49	2.49	6.51	4.63	20	18.97	47.20	32.90

**Table 3:** Microbial analysis of soils.

Parameter	Result (cfu/g)			Method	Conditions
	S1-L	S2-CL	S3-SiCL		
**Aerobic mesophilic bacteria count**	**500,000**	900,000	700,000	IH O:36141	PCA/Aerobic/30 °C/24–72 h
**Proteolytic bacteria**	<10	<10	<10	IH O:36143	PCA-SM/Aerobic/30 °C/24–72 h
**Yeasts**	<50	1500	<50	IH O:43842	RBCA/Aerobic/20.5 °C/3–5 d
**Molds**	3000	15,000	1000	IH O:43842	RBCA/Aerobic/20.5 °C/3–5 d
**Actinomyces**	150	15,000	150	IH:55151	SBA/Anaerobic/35 °C/3–7 d

**Table 4:** Biologic indicators of sandy clay loam soil (S4-SCL) treated with AMI fertilizer.

	Indicator							
VARIANT	Aerobic Cultivable Mesophilic Bacteria Number		Cultivable Fungi Number		Soil Respiration		Microbial Biomass	
	mil cfu/g soil	%	mil cfu/g soil	%	mg CO_2_/100 g soil	%	mg CO_2_/100 g sol	%
**Moist soil (S4-SCL)**	4644	100	6490	100	7977	100	372.8	100
**Hydrogel AMI 0.1%**	7488	161	4722	73	9322	117	445.2	119
**Hydrogel AMI 0.2%**	8416	181	4666	72	9250	91	301.8	81

**Table 5:** Maximum adsorption capacity (A_max_) in mg/g and equilibrium time, hours, of the two soils for nitrogen and phosphorus nutrients released from tested fertilizers.

Soil	Ref-CH				*PSSG*				AMI			
	P		N		P		N		P		N	
	A_max_	t, h	A_max_	t, h	A_max_	t, h	A_max_	t, h	A_max_	t, h	A_max_	t, h
S1-L	1.989	2	6.10	6	0.228	6	13.08	6	0.1105	1	16.50	6
S2-CL	1.775	4	20.17	8	5.700	7	10.80	7	0.9560	7	19.26	4

**Table 6:** Possible structures of the collagen hydrogel fertilizers with various functionalization agents.

Crt.	Functionalization Agents			Possible Structure of the Composite Fertilizers
	Name	Structure	Active Groups	
1	Poly-acryl amide, PAM -synthetic polymers -acrylic resin	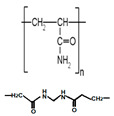 diacrylamide [31]	-NH_2_ (amino groups) >C=O (carbonyl groups)	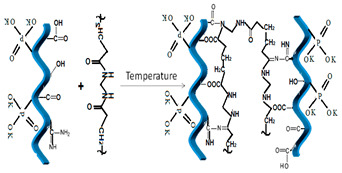
2	Poly(sodium 4-styrenesulfonate-co-glycidyl methacrylate) (P(SSNa-co-GMAx), synthetic polymer	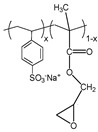 [21]	>C=O (carbonyl groups)	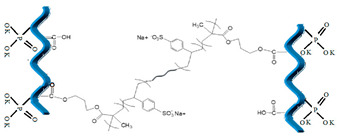
3	Starch, natural polymer	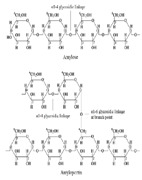 [32]	-OH hydroxyl -CH_2_-O- CH_2-_ glycoside linkages	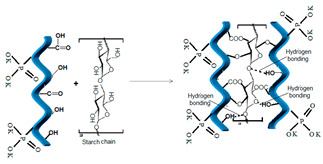
4	Dolomite, natural ore	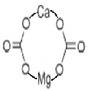 [33]	CO_3_^−^	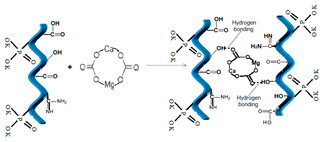

## Data Availability

Not applicable.
